# Incongruence between Cardiorespiratory Fitness and Subjective Reports of Physical Activity in Multiple Sclerosis: A Focus on Sex Differences

**DOI:** 10.1155/2024/6055245

**Published:** 2024-04-24

**Authors:** Syamala Buragadda, Nicholas J. Snow, Alan P. C. Gou, Joshua N. McShane, Caitlin J. Newell, Michelle Ploughman

**Affiliations:** Recovery & Performance Laboratory, Faculty of Medicine, Memorial University of Newfoundland, St. John's, Newfoundland and Labrador, Canada

## Abstract

**Purpose:**

The link between moderate- to vigorous-intensity physical activity (MVPA) and cardiorespiratory fitness in individuals with multiple sclerosis (MS) remains unclear. This study examined the relationship between self-reported MVPA and objectively assessed cardiorespiratory fitness, emphasizing sex differences.

**Methods:**

107 adults with MS (77 females), aged (mean ± standard deviation) 47.2 ± 10.2 years, were recruited from a local MS clinic. Fitness was measured as maximal oxygen uptake (V̇O_2max_) during a graded maximal exercise test using a recumbent stepper. MVPA (24-hour recall) was estimated as the duration of activities ≥ 3 MET (metabolic equivalent of task). MET-minutes were calculated by multiplying MET by duration. We explored sex differences in self-reported MVPA, cardiorespiratory fitness, and disability; examined sex differences in associations between these variables; and investigated whether MET-minutes of MVPA predicted V̇O_2max_ in females and males.

**Results:**

Mean V̇O_2max_ was 24.79 mL·kg^−1^·min^−1^, indicating poor cardiorespiratory fitness levels, despite high levels of self-reported MVPA (mean = 412.5 MET-minutes). Fifty-three percent of males and 40% of females had V̇O_2max_ levels below the 20^th^ age- and sex-standardized population percentile, indicating poor cardiorespiratory fitness. There were statistically significant associations between MVPA and V̇O_2max_ (Rho = 0.27, *p* = .01), as well as disability and V̇O_2max_ (Rho = −0.35, *p* = .02), in females but not males. A regression model using sex, age, body mass, disability, and MVPA to estimate V̇O_2max_ was valid in predicting V̇O_2max_ values that were statistically equivalent to those measured in the laboratory in females but not males. However, the inclusion of MVPA did not add to the predictive value of this equation.

**Conclusions:**

Despite reporting high levels of MVPA, people with MS had poor cardiorespiratory fitness. MVPA, fitness, and disability were associated in females only, indicating that sex differences should be considered in fitness appraisal. Self-reported MVPA did not predict fitness, suggesting 24-hour recall may not be representative of true activity or fitness levels in persons with MS. Future work should examine sex differences in associations between MVPA and fitness using objective measures such as accelerometry.

## 1. Introduction

Multiple sclerosis (MS) is an immune-mediated disease of the central nervous system, characterized by chronic disability accumulation and episodes of new neurologic impairment with incomplete recovery [1]. Among people with MS, vascular comorbidities are associated with accelerated neurodegeneration, earlier disability, and loss of independence [2, 3]. Lifestyle factors are crucial for vascular risk management and the mitigation of disability accumulation [1, 4]. Exercise and physical activity are critical interventions for the promotion of vascular, metabolic, and brain health and should be a routine part of MS care [5–9]. Guidelines recommend that people with MS engage in at least 150 minutes of moderate-to-vigorous physical activity (MVPA) per week [10, 11]. Unfortunately, individuals with MS are less active and more sedentary than healthy controls and even persons with other neurologic disorders like stroke and spinal cord injury [12]. Individuals with MS report disease-related impairments, fatigue, and logistical challenges as barriers to engaging in physical activity [13]. Health professionals cite concerns about patient fatigue and safety as barriers to prescribing physical activity, despite evidence of its safety in MS [14, 15].

One of the first steps in prescribing MVPA is determining the individual's level of fitness. The gold standard cardiorespiratory fitness assessment involves graded maximal exercise testing with indirect calorimetry to measure maximal oxygen uptake (V̇O_2max_) [16]. V̇O_2max_ testing in MS is a valid and reliable measure of aerobic capacity [17] and shows good relationships with disease-specific and general health-related outcomes of the International Classification of Functioning, Disability, and Health model [18]. However, maximal exercise testing and indirect calorimetry require specialized equipment, trained evaluators, and a highly controlled environment. These requirements often preclude maximal exercise testing in real-world clinical or community settings outside the laboratory setting [19]. In some contexts, these limitations can be overcome using submaximal exercise testing or field tests [20]. However, submaximal exercise testing tends to have the greatest validity in low or minimally disabled persons with MS [21, 22], and field test performance may better reflect functional capacity rather than cardiorespiratory fitness or exercise tolerance [22, 23]. When a formal fitness test is impractical, health professionals sometimes rely on patients' subjective reports of physical activity recall. Self-report questionnaires are considered reliable, easy to administer, and more affordable and accessible than fitness testing [24]. In healthy controls, there is good concordance between self-reported physical activity levels, self-appraised fitness, and V̇O_2max_ [25]. However, in MS and other clinical populations, greater susceptibility to recall bias, perceived social desirability, and expectations of others can contribute to the misrepresentation of physical activity levels [26, 27].

MS is a disease with known sex differences, including incidence and onset, disease progression, and the nature and severity of physical and psychosocial impairments [1, 28, 29]. In general, when it comes to reporting cardiorespiratory fitness and physical activity levels among individuals with MS, sex differences are typically overlooked [18, 30]. One study of 92 persons with MS (58 females) found no significant associations between self-reported physical activity and cardiorespiratory fitness (peak V̇O_2_) [31]. However, the authors did not discriminate between different intensities of physical activity nor examine sex differences in physical activity or its association with peak V̇O_2_ [31]. The study sample was recruited from a waiting list of individuals referred for admission to inpatient rehabilitation, so it was likely not representative of people with MS with stable disease who are capable of exercising independently [31]. In another larger study of 380 individuals with MS (249 females), females were less likely to reach V̇O_2max_ before volitional exhaustion compared to males [32]. Also, this study did not compare cardiorespiratory fitness and physical activity levels between the sexes. It is important to note that the study participants were hospital inpatients and may not be representative of independent, community-dwelling individuals. Taken together, these findings allude to the lack of evidence on sex differences in self-reported physical activity levels and cardiorespiratory fitness in MS, highlighting the need for further research to fill existing knowledge gaps.

To address these gaps, the present study is aimed at (1) exploring sex differences in self-reported MVPA and V̇O_2max_; (2) examining relationships between self-reported MVPA, V̇O_2max_, and disability status, with an emphasis on sex differences; and (3) determining whether self-reported MVPA could predict V̇O_2max_ in females and males with MS.

## 2. Materials and Method

### 2.1. Participants

We conducted this cross-sectional study in a neurorehabilitation research laboratory located within a tertiary rehabilitation hospital. Following institutional Health Research Ethics Board approval (HREB#: 2015.103), participants provided informed written consent as per the Declaration of Helsinki. The study sample was recruited from a local MS neurology clinic, and participants were independently able to walk with stable disease.

We recruited consecutive adults diagnosed with MS—using the 2010 or 2017 iterations of the McDonald criteria [33, 34]. We included participants who were aged 18-65 years, had no relapses or new disease activity for ≥3 months, could walk independently with or without gait aides (Expanded Disability Status Scale (EDSS) 0-6) [35], and had no contraindications to exercise [36]. We excluded individuals who screened positive for mild cognitive impairment, scoring ≤ 22 on the Montréal Cognitive Assessment [37]. We extracted EDSS scores and sex assigned at birth from health records.

We planned sample size estimation based on our intention to derive a prediction equation for V̇O_2max_ using participant characteristics and self-reported MVPA. We estimated the target sample size using G∗Power v3.1.9.7 (Aichach, Germany) [38], using data from a recent meta-analysis that suggested sex differences account for up to 36% of the variance in V̇O_2max_ [18]. Based on the coefficient of variation (*R*^2^ = 0.36) and effect size (*f*^2^ = 0.56) gleaned from the study [18], using *α* = .05 and power (1 − *β*) = 0.80) for a multiple linear regression with up to five predictors, we estimated that 54 total participants (27 females, 27 males) would be required to derive a prediction equation for V̇O_2max_. To validate the prediction equation, we estimated that an additional 54 participants (27 females, 27 males) would be required, resulting in a total target sample size of 108. This approach was taken to ensure the validity of the predictive model [39].

### 2.2. Self-Reported MVPA

We asked the participants to recall all activities during the previous 24 hours, describing the details of the activity, duration, and intensity [40]. The 24-hour previous-day recall is a valid tool to estimate active and sedentary behaviors in adults of varying fitness levels (Kozey [40–44]). Previous-day recall methods agree with objective measurements of physical activity, direct observations, and energy expenditure (Kozey [40–42, 44]); and they minimize reporting errors compared to longer-term questionnaires by reducing recall bias due to forgetting (Kozey [40, 41]). Reported activities included sleeping, sitting, walking, activities of daily living, home exercises, and sports, such as running and bicycling. Because of evidence that persons with MS have problems with accurate recall of duration [45], we cleaned self-reported activity data by omitting all values under 10 minutes per day and truncating values over 240 minutes per day [46]. We converted self-reported activities to metabolic equivalents of task (MET) using the 2011 Compendium of Physical Activities [47]. Based on the World Health Organization threshold values, we classified activities with MET ratings > 3.0 METs as MVPA [48]. We calculated MET-minutes of MVPA by multiplying the MET value of each activity by the duration in minutes [48] and reported values for the previous 24 hours.

### 2.3. Cardiorespiratory Fitness

We measured cardiorespiratory fitness using a graded maximal exercise test on a total body recumbent stepper (NuStep T4r, Ann Arbor, MI, USA) [49, 50]. We instructed the participants to avoid alcohol and recreational drugs for ≥24 hours, to avoid caffeine and nicotine for ≥6 hours, and to sleep for ≥6 hours. We measured height (cm), body mass (kg), and body mass index (BMI; kg·m^−2^) with a calibrated device (Health-O-Meter®, McCook, IL, USA), familiarized the participants with the experimental setup, and adjusted the arm and leg attachments of the ergometer based on participant limb length. Participants wore a mask connected to a two-way non-rebreathing valve (Hans Rudolph, Inc., Shawnee, KS, USA). An automated open-circuit indirect calorimetry system with calibrated gas analyzers (Model S-3A and Anarad AR-400; Ametek, Pittsburgh, PA) and tachometer (Model S-430; Vacumetrics/Vacumed Ltd., Ventura, CA) measured expired gas and breathing volumes for breath-by-breath analysis (AEI Technologies, Inc., Pittsburgh, PA, USA). A chest-worn heart rate (HR) monitor transmitted the HR data wirelessly (H10, Polar Electro, Oy, Finland).

Resting blood pressure, V̇O_2_, and HR were measured 5 minutes before exercise. During the test, participants maintained a stepping rate of 80 per minute. The exercise test began at a load level of 3 (20 watts) on a standard scale of 1-10 and increased by 20 watts every 2 minutes. If the participants did not stop by load level 10, we increased the stepping rate by 10 per minute every 2 minutes. Criteria for test termination were (1) volitional exhaustion, (2) inability to maintain workload, or (3) signs of excessive fatigue [49]. We recorded relative V̇O_2_ (normalized to body mass; mL·kg^−1^·min^−1^), HR (bpm), and rating of perceived exertion (RPE; 10 points) [51] at rest before exercise, every 2 minutes during exercise, and after exercise. Participants achieved true V̇O_2max_ if they met two or more of the following criteria: (1) no increase in absolute V̇O_2_ ≥ 150 mL·min^−1^, despite increasing workload; (2) respiratory exchange ratio > 1.10; (3) HR > 90% of the age-predicted maximum; and/or (4) RPE > 8/10 [52]. Besides reporting relative V̇O_2max_, we also reported age- and sex-adjusted percentile ranks of cardiorespiratory fitness per the American College of Sports Medicine (ACSM) [16]. Individuals with a V̇O_2max_ below the 20^th^ percentile for their age and sex have an elevated risk of all-cause mortality [53].

### 2.4. Statistical Analysis

We performed all statistical analyses using SPSS version 27 (IBM Corporation, Armonk, NY, USA). We tested data distributions for normality using the Shapiro-Wilk test and visual inspection of histograms and Q-Q plots. We conducted parametric and nonparametric tests for normal and nonnormal data, respectively. All tests were two-tailed, with the statistical significance threshold at *p* < .05.

Descriptive statistics were reported as proportions (%), mean (standard deviation (SD)), or median (range) for categorical, normal continuous, or nonnormal continuous data, respectively. Sex differences were assessed using parametric (unpaired *t*-test) or nonparametric tests (Mann–Whitney *U*-test or Pearson chi-square test). We estimated effect sizes for *t*-tests using Cohen's *d* with 95% confidence intervals (CI) and interpreted them as trivial (<0.2), small (0.2-0.5), medium (0.5-0.8), and large (≥0.8) [54]. For *U*-tests, we used effect sizes *r* categorized as trivial (<0.1), small (0.1-0.3), medium (0.3-0.5), or large (>0.5) [54]. Chi-square effect sizes were calculated using Cohen's *h* with 95% CI and interpreted as above for Cohen's *d* [54].

We conducted the Spearman Rho (*ρ*) correlations to explore associations between self-reported MVPA, cardiorespiratory fitness, and EDSS, with correlation coefficients interpreted as trivial (<0.1), weak (0.1-0.3), moderate (0.3-0.5), and strong (≥0.5) [54]. Correlations were performed both for the total sample and separately by sex. Sex differences were compared using Fisher *z*-transformations and Cohen's *q*-effect sizes with 95% CI, interpreted as above for Cohen's *d* and *h* [54].

To determine whether self-reported MVPA predicted V̇O_2max_, we performed a multiple linear regression using sex, age, body mass, EDSS, and MET-minutes of MVPA as predictors. These variables were chosen based on their documented contribution to V̇O_2max_ [17, 31] and sex differences in cardiorespiratory fitness [55, 56]. We compared combinations of predictor variables using stepwise linear regression and chose the final model as the combination with the lowest Akaike information criterion (AIC) value. The final model was entered as a standard multiple regression and included each of the above variables—sex, age, body mass, EDSS, and MET-minutes of MVPA. Using a random number generator, we assigned participants to either a regression derivation group (*n* = 50 (34 females, 16 males)) or validation group (*n* = 57 (43 females, 14 males)). The regression equation was derived from the derivation group and validated in the validation group. Groups did not differ significantly in demographics, self-reported physical activity, or V̇O_2max_ (*p* > .05), except for higher EDSS in the validation group (median (range): test group 1.5 (0-6), validation group 2.0 (0-6), *p* = .024). See Predicting V̇O_2max_ from Self-Reported MVPA, under Results, for more information.

We verified the assumption of independence of observations using a Durbin-Watson (DW) statistic of ~2 (DW = 2.056); linearity and homoscedasticity between independent and dependent variables by inspecting plots of unstandardized predicted values versus studentized residuals (*R*^2^ = 1.31 × 10^−5^); and lack of multicollinearity by ensuring Pearson correlations between independent variables were ≥0.7 (Pearson *r* ≤ |0.467|) and variance inflation factors (VIF) were <10 (VIF ≤ 1.382) [57]. There were no outliers (±3 SD from the mean). We confirmed normal distribution of residuals by inspecting histogram and P-P plots for an approximate bell curve and diagonal line, respectively [57]. The model's overall coefficients of variance (*R*^2^ and adjusted *R*^2^) and unstandardized coefficients (*B*, with standard errors) were reported for the derivation group to generate the V̇O_2max_ prediction equation for later validation.

We validated the model using the cross-validation approach [39] and computed predicted V̇O_2max_ values in the validation group using the regression equation from the derivation group [58]. The validity of these estimates was assessed using equivalence testing and Bland-Altman plots [59]. We employed the two one-sided test (TOST) approach to equivalence testing, with paired sample *t*-tests [60]. We set the equivalence threshold (standardized effect size of interest (Cohen's *d*)) at 10% above or below the measured V̇O_2max_ in the derivation group because this is an acceptable margin of error between predicted versus measured V̇O_2max_ in other work that devised V̇O_2max_ prediction equations (Cohen's *d* value of |0.42|) [58]. Nonequivalence was determined if the effect sizes (Cohen's *d*) of measured versus predicted V̇O_2max_ values in the validation group exceeded ±0.42 [60]. Both whole group validation and sex differences in the performance of the regression equation were explored using the TOST approach. We constructed the Bland-Altman plots [59] to assess the degree of error between predicted versus measured V̇O_2max_ and determine the error pattern in females and males [58]. Using this approach, predicted V̇O_2max_ values were considered valid if (1) the difference between, and average of, predicted and measured V̇O_2max_ values was correlated and (2) predicted V̇O_2max_ values fell within 2 SD of measured V̇O_2max_ values [59].

## 3. Results

### 3.1. Participants

Out of 120 participants screened, 13 were excluded due to exercise contraindications [36], leaving 107 individuals in the final sample. The mean ± SD age was 47.2 ± 10.2 years, with a majority being females (*n* = 77 (71.9%)) and 88.8% having relapsing-remitting MS (*n* = 95). The median (range) EDSS was 2.0 (0-6.0). In terms of sex differences, males were significantly taller and heavier (*p* < .001) than females; but other demographic and disease characteristics were not significantly different between sexes (Table 1).

### 3.2. Self-Reported MVPA and Cardiorespiratory Fitness

On average, participants reported engaging in approximately 90 minutes of MVPA (>3.0 METs) in the last 24 hours, accumulating 412.5 MET-minutes. These 24-hour values were close to the recommended weekly 450 MET-minutes of MVPA [61, 62]. Only 10 participants (9.3%) reported no physical activity. The average V̇O_2max_ for participants was 24.80 ± (7.70), placing the median participant in the 10^th^ fitness percentile (poor) [16] (Table 2). Based on the criteria outlined above, 84 participants (78.5%) reached their true V̇O_2max_. For the remaining 23 participants (21.5%), peak V̇O_2_ values are reported as V̇O_2max_.

**Figure 1:**
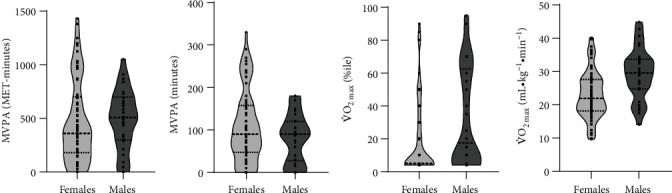
Violin plots illustrating female (light grey) and male (dark grey) moderate- to vigorous-intensity physical activity (MVPA) and cardiorespiratory fitness (maximum oxygen uptake (V̇O_2max_)). Squares are individual data points. Shaded regions represent the distribution of the data. Dashed and dotted lines represent the median and interquartile range (IQR), respectively. (a) Metabolic equivalent of task (MET-minutes of MVPA, (b) minutes of MVPA, (c) age- and sex-normalized V̇O_2max_ percentiles (%ile), and (d) V̇O_2max_ (mL·kg^−1^·min^−1^).

There was no significant difference between males and females regarding self-reported MVPA (*p* > .05) (Table 2 and Figure 1). The proportions of females (*n* = 61 (79.2%)) and males (*n* = 23 (76.7%)) who reached true V̇O_2max_ were not significantly different (*χ*^2^_(1)_ = 0.083, *p* = .773). Males demonstrated a 27% higher relative V̇O_2max_, with a large effect size, compared to females (*p* < .001). When cardiorespiratory fitness was expressed in terms of age- and sex-normalized values, males ranked significantly higher, with a median (range) percentile score of 10 (4-95) versus 5 (4-90) for females and a small effect size (*p* = .026) (Table 2 and Figure 1). Approximately half of both females' and males' cardiorespiratory fitness ranks fell below the 20^th^ percentile.

### 3.3. Associations between MVPA, V̇O_2max_, and Disability

In the total sample, we observed statistically significant positive associations between higher V̇O_2max_ and higher MET-minutes of MVPA (Rho = 0.20, *p* < .05), as well as higher V̇O_2max_ and lower disability (EDSS) (Rho = −0.26, *p* < .01). There was no statistically significant relationship between self-reported MVPA and disability (Rho = −0.10, *p* > .05) (Table 3).

When we analyzed sexes separately, we found a statistically significant yet weak relationship between higher V̇O_2max_ and greater MVPA among females (Rho = 0.27, *p* = .01) but not males (*p* > .05). As well, lower disability (EDSS) was significantly associated with higher V̇O_2max_ in females (Rho = −0.35, *p* = .002) but not males (Rho = −0.20, *p* > .05) (Table 3).

To ascertain whether the lack of statistically significant correlations in males was due to sample size insufficiency, we calculated post hoc sample size requirements based on current sample size (*n* = 30 males), statistical power, correlation coefficients, and *p* values using G∗Power v3.1.9.7 (Aichach, Germany) [38]. To achieve a statistically significant association between MET-minutes of MVPA and relative V̇O_2max_ (power = 0.37, Rho = 0.12, *p* = .280), a target sample size of 185 males would be required. For a statistically significant association between EDSS and relative V̇O_2max_ (power = 0.53, Rho = −0.20, *p* = .290), 102 males would be required. To achieve a statistically significant association between EDSS and percentile ranked V̇O_2max_ (power = 0.64, Rho = −0.11, p = .580), 445 males would be required. Given V̇O_2max_ was significantly associated with both MET-minutes of MVPA EDSS in our sample of 77 females, we interpret this to represent a sex difference, rather than a function of a low sample size of males.

### 3.4. Predicting V̇O_2max_ from Self-Reported MVPA

Thirty-four females and 16 males (*n* = 50) were used to derive the regression equation and 43 females and 14 males (*n* = 57) to validate the equation. Except for a small yet statistically significant difference in EDSS, these groups were not significantly different in terms of demographic or disease characteristics, self-reported MVPA, or objectively measured cardiorespiratory fitness (Table 4).

In the regression derivation group, the overall model was statistically significant (*F*_(5, 49)_ = 6.327, *p* < .001). The combination of sex, age, body mass, EDSS, and MVPA accounted for 35%-42% of variance in V̇O_2max_ (*R*^2^ = 0.418, adjusted *R*^2^ = 0.352) (Table 5). MVPA was the only variable that did not significantly contribute to the predictive ability of the model (*p* > .05). The model met all assumptions. Using the multiple regression, we derived the following equation to analyze sex differences in the prediction of V̇O_2max_: V̇O_2max_ (mL · min^−1^ · kg^−1^) = (8.211 × sex (1 = F, 2 = M)) − (0.228 × age) − (0.247 × body mass (kg))–(0.996 × EDSS) + (0.004 × MET − minutes of MVPA in last 24 hours) + 44.737.

When we ran the regression in the validation group, the overall model remained statistically significant (*F*_(5, 56)_ = 12.989, *p* < .001, *R*^2^ = 0.560, adjusted *R*^2^ = 0.517). Again, MET-minutes of MVPA in the last 24 hours did not reach statistical significance as a predictor variable (*p* > .05). In the validation group, measured and predicted V̇O_2max_ values were both equivalent (*d*(95%CI) = ±0.10 (± -0.16 to +0.36)) and not significantly different (*p* > .05; Table 6).

**Figure 2:**
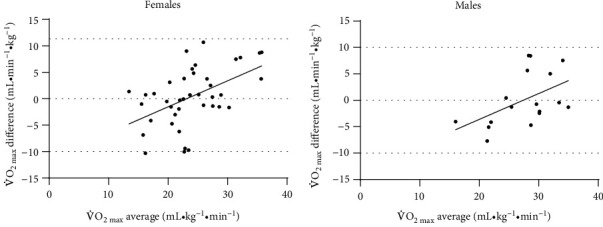
Bland-Altman plots of measured and predicted cardiorespiratory fitness (V̇O_2max_; mL · kg^−1^·min^−1^) in the validation group of participants (*n* = 57; 43 females, 14 males). Based on the regression equation V̇O_2max_ (mL · min^−1^ · kg^−1^) = (8.211 × sex (1 = F, 2 = M)) − (0.228 × age) − (0.247 × body mass) − (0.996 × EDSS) + (0.004 × MET − minutes of MVPA) + 44.737, obtained from the derivation group of participants (*n* = 50; 34 females, 16 males). The *x*-axis represents the average of measured and predicted V̇O_2max_ values, and the *y*-axis represents the difference between measured and predicted values. Dashed lines represent the mean and ±2 standard deviations (SD) from the mean. (a) and (b) demonstrate the prediction equation in females and males, respectively.

When considering sex differences, we found that measured and predicted V̇O_2max_ values were both equivalent (*d* (95%CI) = ±0.001 (± -0.30 to +0.30)) and not significantly different (*p* > .05), in females (Table 6). However, in males, although not significantly different (*p* > .05), the measured and predicted V̇O_2max_ values were also nonequivalent (*d* (95%CI) = ±0.41 (± -0.15 to +0.95); Table 6).  Figure 2 illustrates the Bland-Altman plots of measured and predicted V̇O_2max_ values in females (Figure 2(a)) and males (Figure 2(b)) in the validation group. For both females and males, the difference between, and average of, predicted and measured V̇O_2max_ values was significantly correlated (females: *r* = 0.501, *p* = .001; males: *r* = 0.497, *p* = .042). The plots show that predicted V̇O_2max_ values for all participants fell within 2 SD of measured V̇O_2max_ within both sexes.

## 4. Discussion

This study is aimed at (1) exploring sex differences in self-reported MVPA and V̇O_2max_; (2) examining relationships between self-reported MVPA, V̇O_2max_, and disability status, with an emphasis on sex differences; and (3) determining whether self-reported MVPA could predict V̇O_2max_ in females and males with MS.

MS participants had low levels of cardiorespiratory fitness despite high self-reported levels of MVPA in the last 24 hours, suggesting incongruence between objective fitness levels and self-reported estimates of physical activity. Compared to females, males tended to have greater overall cardiorespiratory fitness, despite similar levels of disability. Next, associations between cardiorespiratory fitness, MVPA, and disability were statistically significant in females only. Lastly, the regression equation including age, sex, body mass, and EDSS and self-reported MET-minutes of MVPA predicted 35%-42% of variance in objectively measured V̇O_2max_; however, self-reported MVPA was the only predictor variable that did not significantly contribute to the equation. The model was valid in females only. We believe these findings suggest that (1) persons with MS tended to overestimate their physical activity levels and (2) 24-hour physical activity recall was not a valid method for estimating cardiorespiratory fitness in persons with MS.

### 4.1. Low Cardiorespiratory Fitness in Males and Females with MS

In the present study, the mean ± SD V̇O_2max_, based on 107 fitness tests conducted on an outpatient MS clinic sample, was 24.80 ± 7.70 mL·kg^−1^·min^−1^, representing fitness in the poor to fair range [16]. Approximately half of all participants had V̇O_2max_ fitness ranks below their age- and sex-normalized 20^th^ percentile [16]. Such low levels of fitness are concerning because of the links between low fitness, metabolic comorbidities, MS disability accumulation, and mortality [2, 3, 53]. A systematic review by Langeskov-Christensen et al. [18] reported similar V̇O_2max_ values in people with MS to those found here, but without considering sex differences [18].

In our sample, despite exceeding recommended physical activity levels based on 24-hour recall self-reports, both females (23.03 ± 7.04 mL·kg^−1^·min^−1^) and males (29.34 ± 7.59 mL·kg^−1^·min^−1^) failed to reach the range of “good” V̇O_2max_ values. There is limited research investigating sex-based differences in physical fitness in MS. A cross-sectional study by Romberg et al. [31] involving 92 individuals with MS (58 females), with a mean age of 44 years, reported fitness values similar to those reported here (21 mL·kg^−1^·min^−1^ for females and 27 mL·kg^−1^·min^−1^ for males) [31]. Interestingly, they reported significant associations between level of disability (EDSS) and fitness, which was stronger in males than females [31]. This finding conflicts with our result that lower disability was associated with higher V̇O_2max_ in females (Rho = −0.35, *p* = .002) but not males (Rho = −0.20, *p* > .05). These differences could be explained by the fact that in the Romberg et al. [31] study, males had higher mean disability scores (EDSS 3.0) than females (EDSS 2.2), while our median EDSS was 2.0 and the same for both sexes. It is important to note that their sample was recruited from a waitlist for inpatient rehabilitation, where participants presumably had rehabilitation needs for walking and balance. Conversely, our sample represents people attending regular outpatient neurology clinic visits, who were not referred to rehabilitation and had independent mobility and whose disease was stable. Given that males tend to have a more severe MS disease course [1], it is possible that their sample was representative of males with severe disease [31].

The method of fitness testing also influences V̇O_2max_ values. Previous studies [31] measured fitness using a cycle leg ergometer. The challenge with using a leg ergometer is that the workload is restricted to the lower limbs, such that individuals with greater leg weakness may not be able to reach their maximal values. Previous research confirmed that MS patients could achieve their predicted maximal fitness values when using both upper and lower body testing but not when using only the arms or legs [20]. In our study, we used a recumbent stepper, a device that has become widely available in the past 15 years and which permits workload distribution between the upper and lower body. Remarkably, even when using a more modern adapted device (recumbent stepper), our group of independent and clinically stable participants had fitness values in the poor to fair range.

### 4.2. Incongruence between Objective Fitness and Self-Reported Physical Activity

Participants reported 90 minutes of MVPA in the last 24 hours (412.5 MET-minutes). For comparison, we were unable to find other studies in MS using 24-hour physical activity recall. In representative MS studies using other self-report instruments, average weekly physical activity levels were variable and included 150 minutes per week of MVPA (≥4 MET) [63], 2710 MET-minutes per week of leisure-time activity of any type and intensity [45], and 1901 MET-minutes per week of at least low-intensity physical activity exceeding (≥3.3 MET) [46]. These observations suggest that participants in the current study tended to overestimate their levels of MVPA using 24-hour recall. Indeed, participants' 24-hour MVPA estimates approached the weekly recommended 450 MET-minutes of MVPA from population physical activity guidelines [61, 62].

Although males tended to report higher levels of MVPA in the previous 24 hours than females (507 MET-minutes vs. 360 MET-minutes), this difference was not statistically significant (*p* > .05). Unlike females, males' self-reported MVPA was not associated with cardiorespiratory fitness. In contrast to our findings, Anens et al. [64] reported lower physical activity levels in males with MS using the Physical Activity Disability Survey (PADS-R). The study suggested that more severe disease in males may limit their physical activity levels to a greater extent than females [64]. Notably, males and females in our sample had similar levels of disability on the neurologist-scored EDSS. Other studies using objective assessments such as uniaxial accelerometry [65] [66] or daily step counts measured by a motion sensor [67] or Fitbit Flex2 device [68] found no sex-related differences among individuals with MS. In a systematic review involving 58 studies, Streber et al. [69] reported that sex was inconsistently associated with physical activity in individuals with MS [69].

Subjective and objective measures of MVPA often show disparities in MS—possibly due to the misinterpretation of activity intensity—which can have significant implications when clinicians evaluate physical activity patterns in individuals with MS [30, 45]. One such source of overrepresentative physical activity self-reporting may be the use of a 24-hour recall instrument. Although these tools have been validated in healthy populations (Kozey [40–44]), previous-day estimates have been shown to misrepresent MVPA due to lack of standardized definitions of activity types and intensity [42–44], for uncommon or unfamiliar activities (Kozey [41]), and for persons with lower fitness [44]. Indeed, potential misclassification of self-reported physical activity in persons with MS can be attributed to a poor understanding or misinterpretation of activity intensity and duration [45]. Kinnett-Hopkins et al. [70] highlighted ambiguities in how individuals with MS perceive and interpret physical activity demands can contribute to challenges in accurately reporting their activity levels [70]. Such challenges are not exclusive to the MS population and have been observed in other chronic conditions such as diabetes [71], rheumatoid arthritis [72], and chronic low back pain [73]. These limitations can be circumvented by using standardized self-report tools that have been validated in the patient population, as well as operationalization of activity descriptions and intensities [74]. Alternatively, objective tools such as accelerometers may provide more valid characterization of physical activity levels [30, 45, 75, 76].

### 4.3. Limitations

One of the limitations of the current study was the self-report questionnaire used to estimate participants' activities in the last 24 hours. We chose the 24-hour recall because of its lower vulnerability to recall bias compared to longer recall periods [26, 27]; however, previous-day estimates of activities may not represent a participant's typical day, especially in persons with MS who may be more vulnerable to inaccurate recall than apparently healthy people. In addition to the timeframe of recall, the process of undertaking an open recall exercise is more nuanced than administering a structured questionnaire. This difference could impact interrater and test-retest reliability of MVPA estimates, thereby reducing the applicability of the present findings to wider clinical practice [45]. Objective measures of physical activity such as accelerometry yield more accurate MVPA results and may better identify sex differences when predicting V̇O_2max_ in future work [30]. Also, we did not explore factors like fatigue, pain, heat sensitivity, comorbidities, lifestyle factors, or medical treatments, nor how they relate to fitness. Since our regression model accounted for less than 50% of the variance in V̇O_2max_, other unmeasured variables may be at play. Future work is needed to reexamine our findings by using other self-report tools or objective measures of MVPA.

## 5. Conclusions

Despite reporting high levels of MVPA, people with MS had low levels of cardiorespiratory fitness. MVPA, fitness, and disability were associated in females only, indicating that sex differences should be considered in fitness appraisal. Self-reported MVPA did not predict fitness, suggesting that 24-hour recall may not be representative of true activity or fitness levels in persons with MS. Low overall levels of fitness point to a need for exercise prescription to promote metabolic and brain health; however, sex should be considered during both fitness appraisal and exercise prescription. Future work should examine sex differences in associations between MVPA and fitness using objective measures such as accelerometry.

## Figures and Tables

**Table 1:** Participant characteristics for the total sample.

Variable	Total (*n* = 107)	Female (*n* = 77)	Male (*n* = 30)	Test statistic	*p* value	Effect size (95% CI)
Age (years) (mean (SD))	47.2 (10.2)	47.3 (9.9)	47.2 (11.2)	*t* = 0.027	.978	*d* = 0.01(-0.42 to +0.43), trivial
Body mass (kg) (median (range))	79.2 (48.0-122.2)	73.9 (48.0-118.3)	86.5 (61.2-122.2)	*U* = 1657.5	<.001^∗^	*r* = 0.34 (0.14-0.53), medium
Height (m) (mean (SD))	1.70 (0.08)	1.67 (0.06)	1.78 (0.07)	*t* = −7.915	<.001^∗^	*d* = −1.70 (-2.18 to -1.22), large
BMI (kg·m^−2^) (median (range))	27.6 (17.9-44.5)	26.8 (17.9-44.5)	27.9 (19.6-40.6)	*U* = 1251.0	.506	*r* = 0.06 (-0.13 to +0.26), trivial
MS type (*n* (%))	RRMS 95 (88.8) PMS 12 (11.2)	RRMS 68 (88.3) PMS 9 (11.7)	RRMS 27 (90.0) PMS 3 (10.0)	*χ* ^2^ = 0.062	.804	*h* = −0.06 (-0.29 to +0.22), trivial
EDSS (median (range))	2.0 (0.0-6.0)	2.0 (0.0-6.0)	2.0 (0.0-6.0)	*U* = 1207.5	.710	*r* = 0.04 (-0.16 to +0.23), trivial

^∗^ *p* < .001. 95% CI: 95% confidence interval; BMI: body mass index; EDSS: Expanded Disability Status Scale; MS: multiple sclerosis; RRMS: relapsing-remitting MS; PMS: progressive MS (including primary and secondary progressive MS).

**Table 2:** Self-reported physical activity and cardiorespiratory fitness.

Variable	Total (*n* = 107)	Female (*n* = 77)	Male (*n* = 30)	Test statistic	*p* value	Effect size (95% CI)
MVPA (minutes) (median (range))	90.0 (0.0-330.1)	90.0 (0.0-330.1)	90.0 (0.0-180.0)	*U* = 989.5	.251	*r* = −0.11 (-0.30 to +0.08), small
MVPA (MET-minutes) (median (range))	412.5 (0.0-1433.6)	360.0 (0.0-1433.6)	507.6 (0.0-1051.5)	*U* = 1322.5	.245	*r* = 0.11 (-0.08 to +0.30), small
V̇O_2max_ (mL·kg^−1^·min^−1^) (mean (SD))^⁋^	24.80 (7.70)	23.03 (7.04)	29.34 (7.59)	*t* = −4.080	<.001^∗∗^	*d* = −0.88 (-1.31 to -0.44), large
V̇O_2max_ (percentile) (median (range))	10 (4-95)	5 (4-90)	17.5 (4-95)	*U* = 1467.0	.026^∗^	*r* = 0.22 (0.02-0.41), small

^∗^ *p* < .05 and ^∗∗^*p* < .001. ^⁋^84 participants (78.5%) reached their true V̇O_2max_. The proportions of females (*n* = 61 (79.2%)) and males (*n* = 23 (76.7%)) who reached true V̇O_2max_ were not significantly different (*χ*^2^_(1)_ = 0.083, *p* = .773). 95% CI: 95% confidence interval; MET: metabolic equivalent of task; MVPA: moderate- to vigorous-intensity physical activity; VO_2max_: peak oxygen uptake.

**Table 3:** Correlations between cardiorespiratory fitness, disability status, and self-reported physical activity.

Variable	Total (*n* = 107)	Female (*n* = 77)	Male (*n* = 30)	Test statistic	*p* value	Effect size (95% CI)
*MVPA (MET-min) (Rho, ρ(95% CI))*						
V̇O_2max_ (mL·kg^−1^·min^−1^)	0.20 (0.00-0.38)^∗^, weak	0.27 (-0.07 to +0.38)^∗^, weak	0.12 (-0.18 to +0.53), weak	*z* = 0.237	.813	*q* = 0.05 (-0.19 to +0.30), trivial
V̇O_2max_ (percentile)	0.24 (0.04-0.41)^∗^, weak	0.22 (-0.01 to +0.43), weak	0.19 (-0.20 to +0.52), weak	*z* = 0.139	.889	*q* = 0.03 (-0.22 to +0.28), trivial
*EDSS (Rho, ρ(95% CI))*						
MVPA (MET-min)	-0.09 (-0.28 to +0.11), trivial	-0.07 (-0.30 to +0.16), trivial	-0.11 (-0.46 to +0.27), weak	*z* = 0.179	.858	*q* = 0.04 (-0.21 to +0.29), trivial
V̇O_2max_ (mL·kg^−1^·min^−1^)	-0.26 (-0.44 to -0.07)^∗^, weak	-0.35 (-0.54 to -0.13)^∗^, moderate	-0.20 (-0.53 to +0.18), weak	*z* = 0.724	.469	*q* = −0.16 (-0.41 to +0.09), small
V̇O_2max_ (percentile)	-0.17 (-0.35 to +0.03), weak	-0.27 (-0.47 to -0.04)^∗^, weak	-0.02 (-0.39 to +0.35), weak	*z* = 1.142	.253	*q* = −0.26 (-0.51 to -0.01), small

^∗^ *p* < .05. 95% CI: 95% confidence interval; EDSS: Expanded Disability Status Scale; MET: metabolic equivalent of task; MVPA: moderate- to vigorous-intensity physical activity; V̇O_2max_: maximum oxygen uptake.

**Table 4:** Comparison of participant characteristics, self-reported physical activity, and cardiorespiratory fitness for regression equation derivation and validation groups.

Variable	Derivation group (*n* = 50)	Validation group (*n* = 57)	Test statistic	*p* value	Effect size (95% CI)
*Participant characteristics*					
Age (years) (mean (SD))	45.8 (10.4)	48.8 (9.8)	*t* = −1.691	.094	*d* = −0.33 (-0.71 to +0.56), small
Sex (*n* (%))	Female 34 (68.0) Male 16 (32.0)	Female 43 (75.4) Male 14 (24.6)	*χ* ^2^ = 0.730	.393	*h* = −0.11 (-0.53 to +0.32), trivial
Body mass (kg) (median (range))	80.3 (48.0-122.2)	76.7 (52.2-118.3)	*U* = 1342.0	.604	*r* = 0.05 (-0.14 to +0.24), trivial
Height (m) (mean (SD))	1.70 (0.09)	1.69 (0.08)	*t* = 0.650	.517	*d* = 0.13 (-0.25 to +0.51), trivial
BMI (kg·m^−2^) (median (range))	27.7 (17.9-40.6)	26.9 (19.7-44.5)	*U* = 1347.0	.626	*r* = 0.05 (-0.14 to +0.24), trivial
MS type (*n* (%))	RRMS 44 (88.0) PMS 6 (12.0)	RRMS 51 (89.5) PMS 6 (10.5)	*χ* ^2^ = 0.058	.810	*h* = −0.03 (-0.18 to +0.15), trivial
EDSS (median (range))	1.5 (0.0-6.0)	2.0 (0.0-6.0)	*U* = 1780.0	.024^∗^	*r* = 0.22 (0.03-0.41), small
*Self-reported physical activity*					
MVPA (minutes) (median (range))	88.5 (0.0-270.0)	90.0 (0.0-330.1)	*U* = 1442.5	.913	*r* = 0.01 (-0.18 to +0.20), trivial
MVPA (MET-minutes) (median (range))	420.0 (0.0-1380.0)	412.5 (0.0-1433.6)	*U* = 1361.5	.692	*r* = 0.04 (-0.15 to +0.23), trivial
*Cardiorespiratory fitness*					
V̇O_2max_ (mL·kg^−1^·min^−1^) (mean (SD))^⁋^	25.30 (7.10)	24.35 (8.23)	*t* = 0.635	.526	*d* = 0.12 (-0.26 to +-.50), trivial
V̇O_2max_ (percentile) (median (range))	10 (4-95)	10 (4-90)	*U* = 1488.0	.685	*r* = 0.04 (-0.15 to +0.23), trivial

^∗^ *p* < .05. ^⁋^The proportions of participants in the regression derivation (*n* = 42 (84.0%)) and validation groups (*n* = 42 (73.7%)) who reached true V̇O_2max_ were not significantly different (*χ*^2^_(1)_ = 1.680, *p* = .195). 95% CI: 95% confidence interval; BMI: body mass index; EDSS: Expanded Disability Status Scale; MET: metabolic equivalent of task; MS: multiple sclerosis; MVPA: moderate- to vigorous-intensity physical activity; RRMS: relapsing-remitting MS; PMS: progressive MS (including primary and secondary progressive MS); V̇O_2max_: maximum oxygen uptake.

**Table 5:** Multiple regression results for objectively measured fitness (V̇O_2max_), based on derivation group.

V̇O_2max_ (mL·kg^−1^·min^−1^)	*B* (95% CI)	SE *B*	*β*	*R* ^2^	Adjusted *R*^2^
Model^∗∗^				0.418	0.352
Constant	44.737 (31.680-57.794)^∗∗^	6.479			
Sex (F, M)	8.211 (4.239-12.184)^∗∗^	1.971	0.545^∗∗^		
Age (years)	-0.228 (-0.394 to -0.062)^∗^	0.083	-0.334^∗^		
Body mass (kg)	-0.247 (-0.379 to -0.115)^∗∗^	0.065	-0.510^∗∗^		
EDSS	-0.996 (-1.914 to -0.078)^∗^	0.455	-0.264^∗^		
MVPA (MET-minutes)	0.004 (-0.002 to +0.009)	0.003	0.168		

^∗^ *p* < .05 and ^∗∗^*p* < .001. 95% CI: 95% confidence interval; *B*: unstandardized regression coefficient; *β*: standardized regression coefficient; EDSS: Expanded Disability Status Scale; MET: metabolic equivalent of task; MVPA: moderate- to vigorous-intensity physical activity; *R*^2^: coefficient of variation; SE *B*: standard error of estimate; V̇O_2max_: maximum oxygen uptake.

**Table 6:** Performance of V̇O_2max_ prediction equation in the validation group.

Measured V̇O_2max_ (mL·kg^−1^·min^−1^) (mean (SD))	Predicted V̇O_2max_ (mL·kg^−1^·min^−1^) (mean (SD))	Effect size (95% CI)	Test statistic	*p* value
*Validation group (n* = 57)				
24.35 (8.23)	23.81 (5.34)	*d* = 0.01 (-0.164 to +0.356)^⁋^, trivial	*t* = 0.038	.970
*Females (n* = 43)				
22.32 (7.56)	22.32 (4.64)	*d* = 0.001 (-0.30 to +0.30)^⁋^, trivial	*t* = −0.790	.434
*Males (n* = 14)				
30.61 (7.16)	28.39 (4.83)	*d* = 0.41 (-0.15 to +0.92), small	*t* = 1.368	.195

^∗^ *p* < .05 and ^∗∗^*p* < .001. ^⁋^Measured and predicted V̇O_2max_ are equivalent. 95% CI: 95% confidence interval; EDSS: Expanded Disability Status Scale; MET: metabolic equivalent of task; MVPA: moderate- to vigorous-intensity physical activity; V̇O_2max_: maximum oxygen uptake.

## Data Availability

Data generated or analyzed during this study are available from the corresponding author upon reasonable request.

## References

[1] Filippi M., Bar-Or A., Piehl F. (2018). Multiple sclerosis. *Nature Reviews Disease Primers*.

[2] Marrie R. A., Patel R., Figley C. R. (2021). Higher Framingham risk scores are associated with greater loss of brain volume over time in multiple sclerosis. *Multiple Sclerosis and Related Disorders*.

[3] Marrie R. A., Rudick R., Horwitz R. (2010). Vascular comorbidity is associated with more rapid disability progression in multiple sclerosis. *Neurology*.

[4] Motl R. W., Sandroff B. M., Kwakkel G. (2017). Exercise in patients with multiple sclerosis. *The Lancet Neurology*.

[5] Dalgas U., Langeskov-Christensen M., Stenager E., Riemenschneider M., Hvid L. G. (2019). Exercise as medicine in multiple sclerosis—time for a paradigm shift: preventive, symptomatic, and disease-modifying aspects and perspectives. *Current Neurology and Neuroscience Reports*.

[6] Devasahayam A. J., Downer M. B., Ploughman M. (2017). The effects of aerobic exercise on the recovery of walking ability and neuroplasticity in people with multiple sclerosis: a systematic review of animal and clinical studies. *Multiple Sclerosis International*.

[7] Devasahayam A. J., Kelly L. P., Williams J. B., Moore C. S., Ploughman M. (2021). Fitness shifts the balance of BDNF and IL-6 from inflammation to repair among people with progressive multiple sclerosis. *Biomolecules*.

[8] Ploughman M., Austin M. W., Glynn L., Corbett D. (2015). The effects of poststroke aerobic exercise on neuroplasticity: a systematic review of animal and clinical studies. *Translational Stroke Research*.

[9] Ploughman M., Kelly L. P. (2016). Four birds with one stone? Reparative, neuroplastic, cardiorespiratory, and metabolic benefits of aerobic exercise poststroke. *Current Opinion in Neurology*.

[10] Kalb R., Brown T. R., Coote S. (2020). Exercise and lifestyle physical activity recommendations for people with multiple sclerosis throughout the disease course. *Multiple Sclerosis Journal*.

[11] Latimer-Cheung A. E., Martin Ginis K. A., Hicks A. L. (2013). Development of evidence-informed physical activity guidelines for adults with multiple sclerosis. *Archives of Physical Medicine and Rehabilitation*.

[12] Kinnett-Hopkins D., Adamson B., Rougeau K., Motl R. W. (2017). People with MS are less physically active than healthy controls but as active as those with other chronic diseases: an updated meta-analysis. *Multiple Sclerosis and Related Disorders*.

[13] Ploughman M. (2017). Breaking down the barriers to physical activity among people with multiple sclerosis – a narrative review. *Physical Therapy Reviews*.

[14] Pilutti L. A., Platta M. E., Motl R. W., Latimer-Cheung A. E. (2014). The safety of exercise training in multiple sclerosis: a systematic review. *Journal of the Neurological Sciences*.

[15] Smith C. M., Hale L. A., Olson K., Baxter G. D., Schneiders A. G. (2013). Healthcare provider beliefs about exercise and fatigue in people with multiple sclerosis. *Journal of Rehabilitation Research and Development*.

[16] Liguori G., Medicine A. C. O. S. (2020). *ACSM's Guidelines for Exercise Testing and Prescription*.

[17] Langeskov-Christensen M., Langeskov-Christensen D., Overgaard K., Møller A. B., Dalgas U. (2014). Validity and reliability of VO_2_-max measurements in persons with multiple sclerosis. *Journal of the Neurological Sciences*.

[18] Langeskov-Christensen M., Heine M., Kwakkel G., Dalgas U. (2015). Aerobic capacity in persons with multiple sclerosis: a systematic review and meta-analysis. *Sports Medicine*.

[19] Hesse C. M., Tinius R. A., Pitts B. C. (2018). Assessment of endpoint criteria and perceived barriers during maximal cardiorespiratory fitness testing among pregnant women. *Journal of Sports Medicine and Physical Fitness*.

[20] Kuspinar A., Andersen R. E., Teng S. Y., Asano M., Mayo N. E. (2010). Predicting exercise capacity through submaximal fitness tests in persons with multiple sclerosis. *Archives of Physical Medicine and Rehabilitation*.

[21] Motl R. W., Fernhall B. (2012). Accurate prediction of cardiorespiratory fitness using cycle ergometry in minimally disabled persons with relapsing-remitting multiple sclerosis. *Archives of Physical Medicine and Rehabilitation*.

[22] Valet M., Lejeune T., Hakizimana J. C., Stoquart G. (2017). Quality of the tools used to assess aerobic capacity in people with multiple sclerosis. *European Journal of Physical and Rehabilitation Medicine*.

[23] Beckerman H., Heine M., van den Akker L. E., de Groot V. (2019). The 2-minute walk test is not a valid method to determine aerobic capacity in persons with multiple sclerosis. *NeuroRehabilitation*.

[24] Mate K. K., Kuspinar A., Ahmed S., Mayo N. E. (2019). Comparison between common performance-based tests and self-reports of physical function in people with multiple sclerosis: does sex or gender matter?. *Archives of Physical Medicine and Rehabilitation*.

[25] Aadahl M., Kjær M., Kristensen J. H., Mollerup B., Jørgensen T. (2008). Self-reported physical activity compared with maximal oxygen uptake in adults. *European Journal of Cardiovascular Prevention & Rehabilitation*.

[26] Cleland C., Ferguson S., Ellis G., Hunter R. F. (2018). Validity of the international physical activity questionnaire (IPAQ) for assessing moderate-to-vigorous physical activity and sedentary behaviour of older adults in the United Kingdom. *BMC Medical Research Methodology*.

[27] Meeus M., van Eupen I., Willems J., Kos D., Nijs J. (2011). Is the international physical activity questionnaire-short form (IPAQ-SF) valid for assessing physical activity in chronic fatigue syndrome?. *Disability and Rehabilitation*.

[28] Shibasaki H., Kuroiwa V. (1976). Sex difference of multiple sclerosis in Japan. *Neurology*.

[29] Zhao Z., Zhang Y., Du Q. (2021). Differences in physical, mental, and social functions between males and females in multiple sclerosis: a multicenter cross-sectional study in China. *Multiple Sclerosis and Related Disorders*.

[30] Motl R. W., Mcauley E., Snook E. M. (2005). Physical activity and multiple sclerosis: a meta-analysis. *Multiple Sclerosis Journal*.

[31] Romberg A., Virtanen A., Aunola S., Karppi S. L., Karanko H., Ruutiainen J. (2004). Exercise capacity, disability and leisure physical activity of subjects with multiple sclerosis. *Multiple Sclerosis Journal*.

[32] Schlagheck M. L., Bansi J., Wenzel C. (2023). Complexity and pitfalls in maximal exercise testing for persons with multiple sclerosis. *European Journal of Neurology*.

[33] Polman C. H., Reingold S. C., Banwell B. (2011). Diagnostic criteria for multiple sclerosis: 2010 revisions to the McDonald criteria. *Annals of Neurology*.

[34] Thompson A. J., Banwell B. L., Barkhof F. (2018). Diagnosis of multiple sclerosis: 2017 revisions of the McDonald criteria. *The Lancet Neurology*.

[35] Kurtzke J. F. (1983). Rating neurologic impairment in multiple sclerosis: an expanded disability status scale (EDSS). *Neurology*.

[36] Bredin S. S., Gledhill N., Jamnik V. K., Warburton D. E. (2013). PAR-Q+ and ePARmed-X+: new risk stratification and physical activity clearance strategy for physicians and patients alike. *Canadian Family Physician*.

[37] Carson N., Leach L., Murphy K. J. (2018). A re-examination of Montreal cognitive assessment (MoCA) cutoff scores. *International Journal of Geriatric Psychiatry*.

[38] Faul F., Erdfelder E., Lang A. G., Buchner A. (2007). G∗Power 3: a flexible statistical power analysis program for the social, behavioral, and biomedical sciences. *Behavior Research Methods*.

[39] Snee R. D. (1977). Validation of regression models: methods and examples. *Technometrics*.

[40] Matthews C. E., Keadle S. K., Sampson J. (2013). Validation of a previous-day recall measure of active and sedentary behaviors. *Medicine and Science in Sports and Exercise*.

[41] Keadle S., Lyden K., Hickey A. (2014). Validation of a previous day recall for measuring the location and purpose of active and sedentary behaviors compared to direct observation. *International Journal of Behavioral Nutrition and Physical Activity*.

[42] Matthews C. E., Berrigan D., Fischer B. (2019). Use of previous-day recalls of physical activity and sedentary behavior in epidemiologic studies: results from four instruments. *BMC Public Health*.

[43] Saraiva Leão Borges L. P., Ries D. C., Sousa A. G., da Costa T. H. M. (2022). Comparison and calibration of 24-hour physical activity recall in adult population. *European Journal of Sport Science*.

[44] Welk G. J., Kim Y., Stanfill B. (2014). Validity of 24-h physical activity recall: physical activity measurement survey. *Medicine and Science in Sports and Exercise*.

[45] Gosney J. L., Scott J. A., Snook E. M., Motl R. W. (2007). Physical activity and multiple sclerosis: validity of self-report and objective measures. *Family & Community Health*.

[46] Marck C. H., Hadgkiss E. J., Weiland T. J., van der Meer D. M., Pereira N. G., Jelinek G. A. (2014). Physical activity and associated levels of disability and quality of life in people with multiple sclerosis: a large international survey. *BMC Neurology*.

[47] Ainsworth B. E., Haskell W. L., Herrmann S. D. (2011). 2011 compendium of physical activities: a second update of codes and MET values. *Medicine and Science in Sports and Exercise*.

[48] Bull F. C., al-Ansari S. S., Biddle S. (2020). World Health Organization 2020 guidelines on physical activity and sedentary behaviour. *British Journal of Sports Medicine*.

[49] Chaves A. R., Kelly L. P., Moore C. S., Stefanelli M., Ploughman M. (2019). Prolonged cortical silent period is related to poor fitness and fatigue, but not tumor necrosis factor, in multiple sclerosis. *Clinical Neurophysiology*.

[50] Kelly L. P., Devasahayam A. J., Chaves A. R. (2017). Intensifying functional task practice to meet aerobic training guidelines in stroke survivors. *Frontiers in Physiology*.

[51] Borg G. (1998). *Borg's Perceived Exertion and Pain Scales*.

[52] Beltz N. M., Gibson A. L., Janot J. M., Kravitz L., Mermier C. M., Dalleck L. C. (2016). Graded exercise testing protocols for the determination of VO_2_max: historical perspectives, progress, and future considerations. *Journal of Sports Medicine*.

[53] Blair S. N., Kohl H. W., Barlow C. E., Paffenbarger R. S., Gibbons L. W., Macera C. A. (1995). Changes in physical fitness and all-cause mortality: a prospective study of healthy and unhealthy men. *JAMA*.

[54] Cohen J. (1988). *Statistical Power Analysis for the Behavioral Sciences*.

[55] Graves R. S., Mahnken J. D., Perea R. D., Billinger S. A., Vidoni E. D. (2015). Modeling percentile rank of cardiorespiratory fitness across the lifespan. *Cardiopulmonary Physical Therapy Journal*.

[56] Lewis D. A., Kamon E., Hodgson J. L. (1986). Physiological differences between genders. Implications for sports conditioning. *Sports Medicine*.

[57] Cohen J., Cohen P., West S. G., Aiken L. S. (2003). *Applied Multiple Regression/Correlation Analysis for the Behavioral Sciences*.

[58] Schembre S. M., Riebe D. A. (2011). Non-exercise estimation of VO_2_ max using the international physical activity questionnaire. *Measurement in Physical Education and Exercise Science*.

[59] Bland J. M., Altman D. G. (1986). Statistical methods for assessing agreement between two methods of clinical measurement. *Lancet*.

[60] Schuirmann D. J. (1987). A comparison of the two one-sided tests procedure and the power approach for assessing the equivalence of average bioavailability. *Journal of Pharmacokinetics and Biopharmaceutics*.

[61] Piercy K. L., Troiano R. P., Ballard R. M. (2018). The physical activity guidelines for Americans. *JAMA*.

[62] Singh R., Pattisapu A., Emery M. S. (2020). US physical activity guidelines: current state, impact and future directions. *Trends in Cardiovascular Medicine*.

[63] Beckerman H., de Groot V., Scholten M. A., Kempen J. C. E., Lankhorst G. J. (2010). Physical activity behavior of people with multiple sclerosis: understanding how they can become more physically active. *Physical Therapy*.

[64] Anens E., Emtner M., Zetterberg L., Hellström K. (2014). Physical activity in subjects with multiple sclerosis with focus on gender differences: a survey. *BMC Neurology*.

[65] Klaren R. E., Motl R. W., Dlugonski D., Sandroff B. M., Pilutti L. A. (2013). Objectively quantified physical activity in persons with multiple sclerosis. *Archives of Physical Medicine and Rehabilitation*.

[66] Fortune J., Norris M., Stennett A. (2021). Correlates of objectively measured physical activity among people with multiple sclerosis: a cross-sectional study. *Frontiers in Rehabilitation Sciences*.

[67] Dlugonski D., Pilutti L. A., Sandroff B. M., Suh Y., Balantrapu S., Motl R. W. (2013). Steps per day among persons with multiple sclerosis: variation by demographic, clinical, and device characteristics. *Archives of Physical Medicine and Rehabilitation*.

[68] Block V. J., Cheng S., Juwono J. (2023). Association of daily physical activity with brain volumes and cervical spinal cord areas in multiple sclerosis. *Multiple Sclerosis Journal*.

[69] Streber R., Peters S., Pfeifer K. (2016). Systematic review of correlates and determinants of physical activity in persons with multiple sclerosis. *Archives of Physical Medicine and Rehabilitation*.

[70] Kinnett-Hopkins D., Learmonth Y., Hubbard E. (2019). The interpretation of physical activity, exercise, and sedentary behaviours by persons with multiple sclerosis. *Disability and Rehabilitation*.

[71] Janevic M. R., Mclaughlin S. J., Connell C. M. (2012). Overestimation of physical activity among a nationally representative sample of underactive Individuals with diabetes. *Medical Care*.

[72] Yu C.-A., Rouse P. C., Veldhuijzen van Zanten J. J. C. S. (2015). Subjective and objective levels of physical activity and their association with cardiorespiratory fitness in rheumatoid arthritis patients. *Arthritis Research & Therapy*.

[73] Schaller A., Rudolf K., Dejonghe L., Grieben C., Froboese I. (2016). Influencing factors on the overestimation of self-reported physical activity: a cross-sectional analysis of low back pain patients and healthy controls. *BioMed Research International*.

[74] Ainsworth B. E., Caspersen C. J., Matthews C. E., Mâsse L. C., Baranowski T., Zhu W. (2012). Recommendations to improve the accuracy of estimates of physical activity derived from self report. *Journal of Physical Activity & Health*.

[75] Boon R. M., Hamlin M. J., Steel G. D., Ross J. J. (2010). Validation of the New Zealand physical activity questionnaire (NZPAQ-LF) and the international physical activity questionnaire (IPAQ-LF) with accelerometry. *British Journal of Sports Medicine*.

[76] Silfee V. J., Haughton C. F., Jake-Schoffman D. E. (2018). Objective measurement of physical activity outcomes in lifestyle interventions among adults: a systematic review. *Preventive Medical Reports*.

